# Health-Related Quality of Life in Women with Uterine Fibroids Recruited from Clinical and Online Settings: A Cross-Sectional Comparative Study

**DOI:** 10.3390/jcm15145657

**Published:** 2026-07-19

**Authors:** Karolina Chmaj-Wierzchowska, Olga Połukord, Oliwia Bajer, Maja Czyżewska, Natalia Handke, Małgorzata Wojciechowska, Małgorzata Piskorz-Szymendera, Maciej Wilczak

**Affiliations:** 1 Department of Maternal and Child Health and Minimally Invasive Gynecologic Surgery, Poznan University of Medical Sciences, 60-701 Poznan, Poland; 2 Laboratory of Advanced Interventional Therapies in Gynecology and Urogynecology, Poznan University of Medical Sciences, 60-701 Poznan, Poland; 3 The Student of Midwifery Program, Faculty of Health Sciences, Poznan University of Medical Sciences, 60-701 Poznan, Poland; 4 Department of Practical Midwifery, Poznan University of Medical Sciences, 41 Jackowskiego Street, 60-512 Poznan, Poland

**Keywords:** uterine fibroids, quality of life, HRQoL, UFS-QoL, online survey, clinic-based survey, women’s health, cross-sectional study

## Abstract

**Background**: Uterine fibroids are the most common benign tumors of the female reproductive system and frequently affect women’s health-related quality of life (HRQoL). The increasing use of online surveys in clinical research raises concerns regarding the comparability and reliability of data collected through different recruitment methods. This study aimed to compare the clinical and methodological comparability of data obtained from an online survey and a clinic-based survey assessing symptom severity and HRQoL among women diagnosed with uterine fibroids. **Methods**: A cross-sectional observational study was conducted among 167 women with confirmed uterine fibroids. The clinic-based cohort included 82 patients recruited in a gynecological outpatient clinic, while the online cohort consisted of 85 women participating through an internet-based survey. Both cohorts were assessed using identical inclusion and exclusion criteria, the same questionnaire structure, and the validated Uterine Fibroid Symptom and Health-Related Quality of Life (UFS-QoL) instrument. Symptom severity scores and HRQoL outcomes were compared descriptively and methodologically. **Results**: The two independently recruited cohorts showed broadly similar distributions of several demographic characteristics and patient-reported outcomes, although important differences between the source populations should be considered when interpreting these findings. Mean symptom severity scores were 48.55 ± 21.61 in the clinic-based cohort and 46.33 ± 21.40 in the online cohort, while mean HRQoL scores were 57.23 ± 22.81 and 62.50 ± 19.32, respectively. Formal comparisons revealed no significant differences in symptom severity (*p* = 0.506) or overall HRQoL (*p* = 0.110). Among the six UFS-QoL domains, only the Concern domain differed significantly between cohorts (*p* = 0.005), whereas all other domains showed broadly similar distributions. Separate multivariable regression models were fitted for each cohort. Although direct comparison of the models was limited by differences in variable coding, both models identified symptom severity as the strongest independent predictor of HRQoL (β = −0.715 and β = −0.752) and showed similar overall model performance (R^2^ = 0.595 and 0.578). Regression diagnostics confirmed normality of residuals, homoscedasticity, absence of influential outliers, and low variance inflation factors in both datasets. **Conclusions**: Despite differences in recruitment setting and source population, similar patterns of patient-reported HRQoL and symptom severity were observed across the two independently recruited cohorts. Similarities were observed across overall HRQoL scores, symptom severity measures, and multivariable regression models, suggesting that the relationships between symptom burden and quality of life remained consistent across recruitment methods. Although differences were identified in disease-related concerns and reproductive history, these findings did not substantially alter the overall pattern of results. Taken together, the findings demonstrate that similar patterns of patient-reported HRQoL and symptom severity were observed across two independently recruited cohorts of women with uterine fibroids. These findings support the feasibility of collecting disease-specific patient-reported outcomes within uterine fibroid-specific online communities but should not be interpreted as evidence that online and clinic-based recruitment methods generate equivalent study populations.

## 1. Introduction

Uterine fibroids are the most common benign tumors affecting women of reproductive age and constitute a major gynecological health problem worldwide [1,2,3,4]. Although many fibroids remain asymptomatic, a substantial proportion of women experience heavy menstrual bleeding, pelvic pain, pressure symptoms, infertility, and impaired quality of life [5,6,7,8]. In recent years, increasing attention has been directed toward the assessment of health-related quality of life (HRQoL) as an essential outcome measure in women with uterine fibroids [9,10]. Traditional clinical parameters such as fibroid size or number do not always correlate with patient-reported symptom burden and psychosocial functioning [11,12,13,14].

The Uterine Fibroid Symptom and Health-Related Quality of Life questionnaire (UFS-QoL) is one of the most widely used disease-specific instruments for evaluating symptom severity and quality of life among women with uterine fibroids [6,7]. Previous studies have demonstrated its good psychometric properties, sensitivity to clinical change, and usefulness in both research and clinical practice [6,7,15].

The rapid expansion of internet-based research has created new opportunities for patient recruitment and data collection. Online surveys enable access to geographically diverse populations and reduce study costs; however, concerns remain regarding representativeness, selection bias, and data reliability [16,17,18,19,20,21,22]. Despite the growing use of online recruitment in studies involving women with uterine fibroids, limited evidence is available regarding whether disease-specific patient-reported symptom severity and health-related quality of life (HRQoL) exhibit similar patterns across independently recruited clinic-based and online cohorts. Therefore, the aim of the present study was to describe and compare UFS-QoL outcomes in two independently recruited cohorts of women with physician-confirmed uterine fibroids rather than to evaluate the equivalence or validity of online versus clinic-based recruitment methods.

## 2. Materials and Methods

### 2.1. Study Design

**Figure 1:**
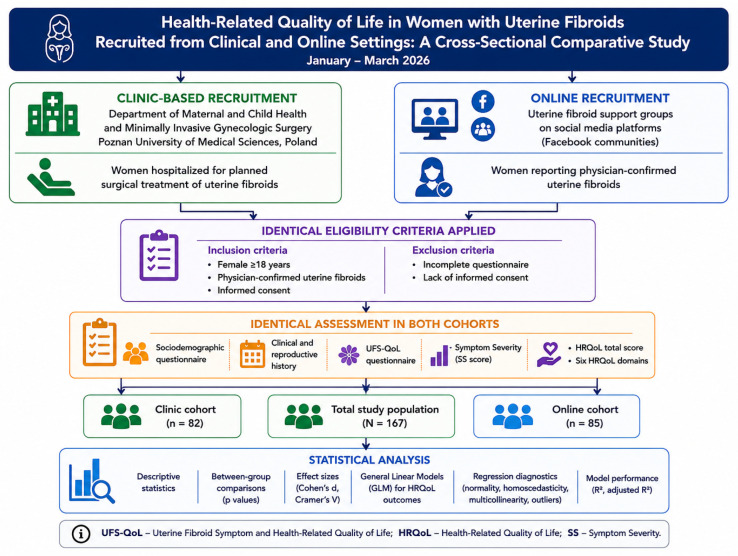
Flowchart of participant recruitment and study design. Women with physician-diagnosed uterine fibroids were recruited either from a tertiary gynecological center (clinic-based cohort) or through disease-specific social media support groups (online cohort). Both cohorts were evaluated using identical eligibility criteria, the same questionnaire structure, and the validated Uterine Fibroid Symptom and Health-Related Quality of Life (UFS-QoL) questionnaire. Data from both recruitment pathways were analyzed using identical statistical procedures.

This study presents a comparative analysis of two independent cross-sectional observational surveys conducted among women diagnosed with uterine fibroids. Both cohorts were assessed using an identical methodological framework, including the same eligibility criteria, questionnaire structure, outcome measures, and statistical procedures. The study employed a diagnostic survey methodology to evaluate symptom severity and health-related quality of life (HRQoL) among women with uterine fibroids. The overall study design and participant recruitment process are presented in Figure 1.

### 2.2. Participants

The clinic-based cohort was recruited at the Department of Maternal and Child Health and Minimally Invasive Gynecologic Surgery, Heliodor Święcicki Clinical Hospital, Poznan University of Medical Sciences. The study included 82 women with a physician-confirmed diagnosis of uterine fibroids who were hospitalized for planned surgical treatment between January and March 2026.

The online cohort consisted of 85 women recruited between January and March 2026 through disease-specific Facebook support groups dedicated to women with uterine fibroids. Invitations to participate included a brief description of the study together with a link to a self-administered questionnaire created using Google Forms (Google LLC., Mountain View, CA, USA). Participation was voluntary and anonymous. Before accessing the questionnaire, participants were informed about the study objectives and provided electronic informed consent. Only questionnaires that were completed in full and met the predefined eligibility criteria were included in the final analyses, whereas incomplete questionnaires were excluded. Because the survey was anonymous, duplicate participation could not be completely excluded. However, all questionnaires were screened before analysis for completeness, logical consistency, and potential duplicate records based on response patterns, and no obvious duplicate submissions were identified.

For both cohorts, inclusion criteria comprised female sex, age ≥ 18 years, and a confirmed diagnosis of uterine fibroids. Exclusion criteria included incomplete questionnaire responses and lack of informed consent. Identical eligibility criteria were applied to maximize comparability between the study populations.

### 2.3. Research Instrument

Data were collected using a structured questionnaire entitled “Quality of Life Assessment in Women with Uterine Fibroids”. The survey consisted of 41 questions divided into three main sections: sociodemographic characteristics, clinical and reproductive history, and quality-of-life assessment. The sociodemographic section included questions regarding age, place of residence, marital status, educational level, occupational status, and socioeconomic conditions. Clinical and reproductive variables included time since diagnosis of uterine fibroids, gynecological follow-up, treatment history, medication use, reproductive history, fertility problems, childbirth history, and lifestyle-related factors such as physical activity and dietary habits. The questionnaire also assessed the impact of uterine fibroids on multiple dimensions of daily functioning. Respondents reported physical symptoms, including heavy menstrual bleeding, pelvic pain, pressure symptoms, urinary urgency, and constipation. Additional questions evaluated limitations in daily activities, occupational functioning, sexual health, emotional well-being, self-perception, and social support. The survey further explored psychological consequences of the disease, including anxiety, stress, depressive symptoms, fatigue, perceived loss of control, self-esteem, and feelings of embarrassment associated with fibroid-related symptoms.

Health-related quality of life was additionally assessed using the validated Uterine Fibroid Symptom and Health-Related Quality of Life questionnaire (UFS-QoL), developed by Spies et al. [6] and further validated by Coyne et al. [7]. The instrument measures symptom severity and six quality-of-life domains: Concern, Activities, Energy/Mood, Control, Self-Consciousness, and Sexual Function. Higher scores on the symptom severity scale indicate greater symptom burden, whereas higher scores in the HRQoL domains reflect better quality of life.

The same questionnaire structure, eligibility criteria, and outcome measures were used in both the online and clinic-based cohorts to ensure methodological comparability between the two study populations.

### 2.4. Statistical Analysis

Data analysis was performed using Statistica 14, Jamovi 2.3, and Microsoft Excel. In both studies, distribution normality was evaluated using the Shapiro–Wilk test. Comparative analyses of continuous variables between cohorts were conducted using the Mann–Whitney U test or the independent-samples *t*-test, as appropriate, with effect sizes reported as Cohen’s d; categorical variables were compared using the chi-square test, with effect sizes reported as Cramér’s V. Multivariable analyses employed General Linear Models (GLMs). Variance Inflation Factors (VIFs) were calculated to assess multicollinearity, and statistical significance was established at *p* < 0.05.

Multivariable General Linear Models (GLMs) were constructed separately for the clinic-based and online cohorts, using overall HRQoL score as the dependent variable. Independent variables included symptom severity score together with selected sociodemographic and clinical characteristics (age, education level, place of residence, marital status, employment status, parity, time since diagnosis, and cohort-specific clinical variables). Categorical variables were entered using dummy coding. Predictor selection was based on clinical relevance and data availability within each cohort. Model assumptions were assessed by examination of residual distributions, homoscedasticity, influential observations, and variance inflation factors (VIFs).

Comparisons of individual UFS-QoL domains were considered exploratory secondary analyses. Therefore, no formal adjustment for multiple comparisons was applied, and these findings should be interpreted cautiously.

## 3. Results

The study included 167 women diagnosed with uterine fibroids. Of these, 82 participants were recruited from a clinical setting, while 85 respondents were enrolled through an online survey. Age comparability between cohorts could not be formally assessed because age was collected and reported using different formats. In the clinic-based cohort, age was summarized as a median value (45 years), whereas in the online cohort, age was recorded in predefined categories. Among online respondents, 78.9% were aged 36 years or older, 13.3% were aged 30–35 years, 6.7% were aged 24–29 years, and 1.1% were aged 18–23 years. Because individual age data were not available in a comparable format, formal statistical testing of age differences between cohorts was not possible. This limitation should be considered when interpreting differences in parity, fertility history, symptom burden, and health-related quality of life.

### 3.1. Characteristics of the Study Population

The difference in sample size between the two groups was minimal (3.6%), which limits differences in statistical precision (e.g., confidence interval width) between cohorts but does not by itself imply similarity of the underlying source populations. Women with higher education constituted 76.8% of the clinic-based cohort and 67.1% of the online cohort. Rural residents accounted for 30.5% and 28.2% of participants, respectively, while married women represented approximately two-thirds of both cohorts (64.6% and 62.4%, respectively). Overall, the distributions of educational level, place of residence, and marital status were broadly similar, although differences in parity and age reporting should be considered when interpreting these findings. The online cohort included a higher proportion of women with previous childbirth, which may be related to differences in age structure and disease duration rather than recruitment setting itself (Table 1).

### 3.2. Symptom Severity

Symptom severity scores demonstrated a high degree of consistency across recruitment methods. In the clinic-based cohort, the mean symptom severity score was 48.55 ± 21.61, compared with 46.33 ± 21.40 in the online cohort. Median values were 53.13 and 46.15, respectively. The 95% confidence intervals showed considerable overlap (43.8–53.3 vs. 41.72–50.95), suggesting similar observed symptom severity between the two populations. The small difference between mean values (2.22 points on the 0–100 scale) indicates only modest observed differences in symptom severity between the two independently recruited cohorts (Table 2).

### 3.3. Health-Related Quality of Life

The mean overall HRQoL score was 57.23 ± 22.81 in the clinic-based cohort and 62.50 ± 19.32 in the online cohort. Median values were 60.34 and 65.79, respectively. Although the online cohort achieved slightly higher HRQoL scores, the confidence intervals overlapped substantially (52.22–62.25 vs. 58.34–66.67), indicating only modest observed differences in HRQoL between the two independently recruited cohorts (Table 3).

### 3.4. Comparison of UFS-QoL Domains

The distribution of scores across the six UFS-QoL domains was similar in both cohorts. Mean values ranged between approximately 50 and 70 points in all domains. In the clinic-based cohort, the lowest scores were observed in the domains of Concern (51.52 ± 30.08) and Sexual Function (52.29 ± 28.74). A similar pattern was observed in the online cohort, where Sexual Function (57.65 ± 30.87) and Energy/Mood (53.53 ± 25.47) demonstrated the lowest scores. The highest scores were recorded in Self-Consciousness (68.47 ± 34.14) and Control (66.18 ± 31.51) in the online cohort and in Energy/Mood (59.89 ± 22.84) and Activities (57.67 ± 25.48) in the clinic-based cohort. Overall, both cohorts demonstrated a similar ranking of HRQoL domains, indicating a similar pattern of disease impact across multiple dimensions of daily functioning (Table 4).

### 3.5. Multivariable Regression Analysis

Both regression models demonstrated strong overall performance. In the clinic-based cohort, the model explained 59.5% of the variance in HRQoL (R^2^ = 0.595; adjusted R^2^ = 0.543), whereas in the online cohort, the corresponding values were 57.8% and 53.4%, respectively. Symptom severity emerged as the strongest independent predictor of HRQoL in both cohorts. Standardized regression coefficients were highly similar (β = −0.715 and β = −0.752), indicating a strong inverse relationship between symptom burden and quality of life. The magnitude of effect was also similar, with partial eta-squared values exceeding 0.50 in both models, suggesting that symptom severity accounted for more than half of the explained variance in HRQoL. Sociodemographic variables showed limited influence on HRQoL after adjustment for clinical factors. Their effect sizes remained small and did not materially affect the association between symptom severity and quality of life.

The clinic-based model explained 59.5% of the variance in HRQoL (R^2^ = 0.595; adjusted R^2^ = 0.543; F(9,70) = 11.44; *p* < 0.001), whereas the online model explained 57.8% (R^2^ = 0.578; adjusted R^2^ = 0.534; F(8,76) = 13.01; *p* < 0.001). Regression diagnostics confirmed approximate normality of residuals, homoscedasticity, absence of influential observations, and low multicollinearity in both cohorts (VIF range: 1.10–1.50 for the clinic-based cohort and 1.12–1.78 for the online cohort). Symptom Severity was the strongest independent predictor of HRQoL in both models (β = −0.715 and β = −0.752, respectively; both *p* < 0.001). Time since diagnosis remained an additional independent predictor only in the online cohort (β = −0.213, *p* = 0.010), whereas none of the remaining sociodemographic or clinical variables reached statistical significance after adjustment (Table 5).

### 3.6. Comparison Between Recruitment Methods

To evaluate the consistency of findings obtained through different recruitment strategies, descriptive comparisons were performed between the clinic-based and online cohorts. Mean symptom severity scores were similar between groups (48.55 ± 21.61 vs. 46.33 ± 21.40), as were overall HRQoL scores (57.23 ± 22.81 vs. 62.50 ± 19.32). Substantial overlap of the corresponding 95% confidence intervals was observed for both primary outcomes. Across the six UFS-QoL domains, mean values remained within similar ranges in both cohorts. The lowest scores were consistently observed in domains related to disease concerns and sexual functioning, whereas higher scores were observed in domains reflecting daily functioning, self-awareness, and perceived control. Furthermore, multivariable regression analyses yielded highly similar results in both cohorts. Symptom severity was identified as the strongest predictor of HRQoL, with standardized regression coefficients of β = −0.715 and β = −0.752, respectively. The explanatory power of the regression models was also comparable (R^2^ = 0.595 vs. 0.578), supporting the consistency of associations observed across recruitment methods.

To further evaluate the comparability of recruitment methods, formal between-group analyses were performed. No statistically significant differences were observed between the clinic-based and online cohorts regarding overall symptom severity scores (*p* = 0.506, Cohen’s d = 0.10) or overall HRQoL scores (*p* = 0.110, Cohen’s d = −0.25). Similarly, no significant differences were found for educational level (*p* = 0.160), place of residence (*p* = 0.749), or marital status (*p* = 0.760), with all effect sizes remaining small (Cramér’s V < 0.11).

Across the six UFS-QoL domains, only the Concern domain demonstrated a statistically significant difference between cohorts (*p* = 0.005, Cohen’s d = −0.44), indicating moderately higher scores among online respondents. Differences in Activities (*p* = 0.056), Energy/Mood (*p* = 0.091), Control (*p* = 0.112), Self-Consciousness (*p* = 0.061), and Sexual Function (*p* = 0.247) did not reach statistical significance. Among demographic variables, parity differed significantly between cohorts (*p* = 0.011, Cramér’s V = 0.20), with a higher proportion of women reporting previous childbirth in the online cohort. Overall, these findings indicate broadly similar patient-reported outcome patterns, while also highlighting differences in reproductive history and disease-related concerns (Table 6).

## 4. Discussion

The present study demonstrated substantial, though not absolute, agreement between data obtained through online and clinic-based recruitment strategies. Across both cohorts, symptom severity scores, overall HRQoL values, and domain-specific UFS-QoL outcomes exhibited largely comparable distributions, despite differences in the recruitment environment, with the exception of the Concern domain and parity, which differed significantly between cohorts and are discussed below. These findings indicate that similar patterns of patient-reported symptom severity and HRQoL were observed across the two independently recruited cohorts, although the underlying populations differed in several important clinical and demographic characteristics. Detailed imaging characteristics, including FIGO classification and fibroid location, were not available for participants recruited online. Therefore, subgroup analyses according to fibroid location were beyond the scope of the present comparative study. Differences in age structure, reproductive history, and disease duration between the two cohorts should be considered when interpreting the observed findings. Because these variables are closely related to the clinical presentation and treatment pathway of uterine fibroids, it cannot be determined whether the observed similarities and differences reflect recruitment setting, underlying population characteristics, or a combination of both factors.

Similar observations have been reported in studies evaluating the validity of internet-based health research. Previous authors have demonstrated that online surveys can generate reliable patient-reported outcome measures, particularly when recruitment criteria, questionnaire structure, and analytical procedures are carefully standardized [21,22,23,24,25]. The increasing accessibility of digital technologies has transformed epidemiological and clinical research, allowing investigators to reach geographically dispersed populations while substantially reducing organizational costs and participant burden [20,21,22,23,24].

Online surveys have become especially valuable in studies involving chronic diseases, where long-term symptom monitoring and quality-of-life assessment are essential. Women affected by uterine fibroids often experience persistent symptoms that influence multiple dimensions of daily functioning, including occupational activity, social relationships, emotional well-being, and sexual health [4,5]. Consequently, patient-reported outcome measures represent an indispensable complement to traditional clinical assessment, provided they meet established standards of content validity and methodological rigor [23,24]. Disease-specific instruments such as the UFS-QoL provide a more comprehensive understanding of the burden of uterine fibroids than generic health status measures, such as the SF-36 [25], or objective clinical parameters alone, including fibroid size, number, or anatomical location [6,11].

One of the most important findings of the present study was the similar descriptive patterns observed in symptom severity scores between cohorts. Mean symptom severity values differed by only 2.22 points on the 0–100 scale, while confidence intervals showed substantial overlap. This observation indicates that similar levels of symptom severity were observed in the two independently recruited cohorts. Importantly, the consistency of findings was not limited to symptom severity alone. Overall HRQoL scores demonstrated a similar pattern, with only modest differences between cohorts despite recruitment occurring in substantially different settings.

The slightly higher HRQoL scores observed among online respondents may reflect differences in disease adaptation rather than methodological bias. Women recruited through social media support groups included a greater proportion of participants with a longer disease duration. Previous research has shown that adaptation to chronic illness may improve coping strategies, disease acceptance, and perceived quality of life despite persistent symptoms [5,26]. One possible explanation is that women with a longer disease duration may have developed greater familiarity with their condition and different patterns of adaptation over time. However, because psychological adaptation, coping strategies, and disease acceptance were not measured, this interpretation remains speculative. An equally plausible explanation is that women recruited through disease-specific online communities differed from the clinic-based cohort with respect to unmeasured characteristics related to health-seeking behavior, disease perception, or participation in online support groups. Consequently, the present cross-sectional study cannot distinguish between these alternative explanations. Among the six UFS-QoL domains, only the Concern domain demonstrated a statistically significant difference between cohorts. However, because analyses of individual domains were predefined exploratory secondary analyses and no formal correction for multiple comparisons was applied, this finding should be interpreted with appropriate caution. It may represent a true difference between the cohorts, but it should also be considered hypothesis-generating and requires confirmation in future studies specifically designed to evaluate domain-level differences. This finding is consistent with previous reports indicating that uterine fibroids exert a substantial psychosocial impact extending beyond physical symptoms [4,12]. Concerns regarding disease progression, fertility, treatment outcomes, and future health frequently contribute to psychological distress among affected women. Likewise, impaired sexual functioning has been repeatedly identified as one of the most vulnerable dimensions of quality of life in women with symptomatic uterine fibroids [4,5]. Notably, the Concern domain was the only domain to differ significantly between cohorts, with online respondents reporting moderately higher (i.e., better) scores than clinic-based participants. Because this domain captures worries about disease progression, fertility, and future health, this difference may relate to the longer disease duration and greater representation of parous women observed in the online cohort, consistent with the disease-adaptation hypothesis discussed above, although selection effects associated with recruitment through disease-specific support groups cannot be excluded as an alternative explanation.

Another important observation was that the separate multivariable regression models yielded similar descriptive patterns in both cohorts. In each model, symptom severity emerged as the strongest independent predictor of HRQoL, with similar standardized regression coefficients (β = −0.715 and β = −0.752, respectively) and comparable model performance (R^2^ = 0.595 and 0.578, respectively). Regression diagnostics confirmed normality of residuals, homoscedasticity, absence of influential observations, and low multicollinearity in both models. However, because the models were fitted separately and included variables that were not fully harmonized across cohorts, these findings should be interpreted descriptively rather than as evidence of equivalent associations between recruitment settings. Overall, the analyses indicate that symptom severity was consistently associated with HRQoL in both independently recruited cohorts, while acknowledging the methodological limitations inherent to the study design.

From a practical perspective, these results have important implications for future gynecological research. Online recruitment may facilitate access to populations that are difficult to reach through conventional clinical pathways, including women living in rural areas, individuals receiving treatment in multiple healthcare settings, and patients actively seeking disease-related information and support. Given the increasing emphasis on patient-centered care and patient-reported outcomes, digital recruitment strategies may become an increasingly important component of future observational and epidemiological studies.

Nevertheless, online recruitment should not be considered a complete replacement for clinic-based research. Clinical studies provide access to objective diagnostic information, treatment details, and physician-confirmed outcomes that may not always be available through self-reported questionnaires. Therefore, the most effective approach may involve combining traditional clinical recruitment with carefully designed online data collection strategies, thereby maximizing both scientific rigor and population reach.

Overall, the present findings suggest that carefully designed online surveys can provide useful disease-specific patient-reported outcome data within uterine fibroid-specific online communities. However, because the study compared two independently recruited source populations, the findings should not be interpreted as evidence that online and clinic-based recruitment methods generate equivalent study populations. Further studies involving more representative populations, standardized clinical data collection, and harmonized participant characteristics are needed to better understand the influence of recruitment setting on patient-reported outcomes. Extension of these findings to other gynecological conditions, recruitment platforms, or populations requires confirmation in independent studies.

## 5. Limitations

Several limitations should nevertheless be acknowledged. First, the cross-sectional design precludes causal inference, and the absence of random allocation does not allow complete elimination of residual confounding [27].

Second, important limitations relate to the online recruitment strategy. Participants were recruited through disease-specific social media support groups, which may have preferentially attracted women who were more engaged with their condition, more motivated to seek information, or experiencing a different disease burden than the general population of women with uterine fibroids. Consequently, the findings cannot be generalized to all women with uterine fibroids or to online populations beyond similar disease-specific communities. In addition, the diagnosis of uterine fibroids in the online cohort relied on self-reported physician confirmation and was not independently verified using medical records or imaging documentation. Although incomplete questionnaires were excluded and responses were screened for consistency, self-reported outcomes remain susceptible to reporting bias, and the possibility of diagnostic misclassification cannot be completely excluded.

Third, several clinically relevant variables were unavailable in the online cohort. Objective imaging characteristics, including fibroid size, fibroid number, and FIGO classification, were not collected because standardized ultrasound documentation was unavailable. Likewise, concomitant gynecological conditions, particularly adenomyosis and endometriosis, were not systematically assessed. As these conditions may independently influence symptom severity and health-related quality of life, residual clinical confounding cannot be excluded.

Fourth, age represented an important methodological limitation. Age was collected using different formats in the two cohorts (continuous values in the clinic-based cohort and predefined age categories in the online cohort), preventing formal comparison of age distributions and requiring different coding strategies in the multivariable regression models. Consequently, the regression models were not fully equivalent, and similarities between them should be interpreted with caution. Moreover, the higher proportion of parous women in the online cohort may reflect differences in age structure and disease duration rather than recruitment setting itself.

Finally, analyses of individual UFS-QoL domains were predefined exploratory secondary analyses, and no formal correction for multiple comparisons was applied. Therefore, isolated statistically significant findings, particularly those concerning individual HRQoL domains, should be interpreted cautiously and considered hypothesis-generating until confirmed in independent studies.

## 6. Conclusions

Despite differences in recruitment setting and source population, similar patterns of patient-reported HRQoL and symptom severity were observed across the two independently recruited cohorts. Similarities were observed across overall HRQoL scores, symptom severity measures, and multivariable regression models, suggesting that the relationships between symptom burden and quality of life remained consistent across recruitment methods. Although differences were identified in disease-related concerns and reproductive history, these findings did not substantially alter the overall pattern of results. Taken together, the findings demonstrate that similar patterns of patient-reported HRQoL and symptom severity were observed across two independently recruited cohorts of women with uterine fibroids. These findings support the feasibility of collecting disease-specific patient-reported outcomes within uterine fibroid-specific online communities but should not be interpreted as evidence that online and clinic-based recruitment methods generate equivalent study populations.

## Figures and Tables

**Table 1:** Demographic characteristics of the study population according to recruitment method.

Characteristic	Clinic-Based Survey (*n* = 82)	Online Survey (*n* = 85)
Sample size, *n*	82	85
Higher education, *n* (%)	63 (76.8)	57 (67.1)
Residence		
Rural, *n* (%)	25 (30.5)	24 (28.2)
Urban, *n* (%)	57 (69.5)	61 (71.8)
Marital status		
Married, *n* (%)	53 (64.6)	53 (62.4)
Other, *n* (%)	29 (35.4)	32 (37.6)
Parity		
Women with previous childbirth, *n* (%)	39 (48.8)	57 (67.1)
Nulliparous women, *n* (%)	43 (51.2)	28 (32.9)
Predominant age group	Median age: 45 years	≥36 years (78.9%)

**Table 2:** Comparison of symptom severity scores between the clinic-based and online cohorts.

Parameter	Clinic-Based Survey (*n* = 82)	Online Survey (*n* = 85)
Minimum	0.00	7.69
Maximum	84.38	100.00
Mean ± SD	48.55 ± 21.61	46.33 ± 21.40
Median	53.13	46.15
95% Confidence Interval	43.80–53.30	41.72–50.95
Range of overlap between the two 95% CIs above	43.80–50.95	

**Table 3:** Comparison of overall health-related quality of life (HRQoL) scores between the clinic-based and online cohorts.

Parameter	Clinic-Based Survey (*n* = 82)	Online Survey (*n* = 85)
Minimum	6.03	15.79
Maximum	100.00	100.00
Mean ± SD	57.23 ± 22.81	62.50 ± 19.32
Median	60.34	65.79
95% Confidence Interval	52.22–62.25	58.34–66.67
Range of overlap between the two 95% CIs above	58.34–62.25	58.34–62.25

Abbreviations: HRQoL—health-related quality of life; SD—standard deviation; CI—confidence interval.

**Table 4:** Comparison of UFS-QoL domain scores between the clinic-based and online cohorts.

UFS-QoL Domain	Clinic-Based Survey (*n* = 82) Mean ± SD	Online Survey (*n* = 85) Mean ± SD
Concern	51.52 ± 30.08	62.88 ± 20.47
Activities	57.67 ± 25.48	65.06 ± 23.99
Energy/Mood	59.89 ± 22.84	53.53 ± 25.47
Control	59.33 ± 23.45	66.18 ± 31.51
Self-Consciousness	59.35 ± 28.25	68.47 ± 34.14
Sexual Function	52.29 ± 28.74	57.65 ± 30.87

Abbreviations: UFS-QoL—Uterine Fibroid Symptom and Health-Related Quality of Life questionnaire; SD—standard deviation.

**Table 5:** Multivariable linear regression models predicting health-related quality of life (HRQoL) in the clinic-based and online cohorts.

Predictor	Clinic-Based Cohort (*n* = 82)					Online Cohort (*n* = 85)				
	B	SE	β	t	*p*	B	SE	β	t	*p*
Intercept	1.06	0.19	—	5.62	<0.001	0.93	0.04	—	21.10	<0.001
Age	0.00	0.00	0.02	0.25	0.81	−0.02	0.02	−0.06	−0.71	0.48
Education	0.00	0.02	−0.01	−0.18	0.86	0.01	0.02	0.03	0.39	0.69
Residence	−0.02	0.02	−0.10	−1.07	0.29	0.00	0.02	0.02	0.28	0.78
Marital status	−0.02	0.02	−0.08	−0.95	0.34	0.01	0.02	0.05	0.59	0.56
Employment status	—	—	—	—	—	−0.03	0.02	−0.11	−1.40	0.17
BMI	−0.01	0.01	−0.11	−1.44	0.15	—	—	—	—	—
Menstrual duration	0.02	0.01	0.12	1.36	0.18	—	—	—	—	—
Menstrual bleeding severity	−0.02	0.01	−0.17	−1.78	0.08	—	—	—	—	—
Time since diagnosis	—	—	—	—	—	−0.04	0.02	−0.21	−2.61	0.010
Parity	0.01	0.02	0.04	0.50	0.62	0.01	0.02	0.04	0.45	0.65
Symptom Severity score	−0.75	0.08	−0.72	−8.80	<0.001	−0.68	0.07	−0.75	−9.51	<0.001
95% CI for Symptom Severity coefficient	−0.877 to −0.553					−0.820 to −0.530				

**Abbreviations:** B, unstandardized regression coefficient; SE, standard error; β, standardized regression coefficient; HRQoL, health-related quality of life.

**Table 6:** Formal comparison of clinic-based and online cohorts.

Variable	*p*-Value	Effect Size
Symptom Severity Score	0.506	Cohen’s d = 0.10
Overall HRQoL	0.110	Cohen’s d = −0.25
Concern	0.005	Cohen’s d = −0.44
Activities	0.056	Cohen’s d = −0.30
Energy/Mood	0.091	Cohen’s d = 0.26
Control	0.112	Cohen’s d = −0.25
Self-Consciousness	0.061	Cohen’s d = −0.29
Sexual Function	0.247	Cohen’s d = −0.18
Higher education	0.160	Cramér’s V = 0.11
Rural residence	0.749	Cramér’s V = 0.03
Married	0.760	Cramér’s V = 0.02
Previous childbirth	0.011	Cramér’s V = 0.20

## Data Availability

The data presented in this study are available on request from the corresponding authors.
